# Challenges in the management of acute intermittent porphyria with recurrent attacks during pregnancy: A case report

**DOI:** 10.1002/ccr3.3185

**Published:** 2020-07-30

**Authors:** Daphne Vassiliou, Chariklia Lempessi, Pauline Harper, Eliane Sardh

**Affiliations:** ^1^ Department of Endocrinology and Nephrology, Inflammation and Infection Karolinska University Hospital Stockholm Sweden; ^2^ Centre for Inherited Metabolic Diseases (CMMS) Porphyria Centre Sweden Karolinska University Hospital Stockholm Sweden; ^3^ Department of Molecular Medicine and Surgery Karolinska Institutet Stockholm Sweden; ^4^ Department of Medicine Örebro University Hospital Örebro Sweden; ^5^ Department of Medical Biochemistry and Biophysics Karolinska Institutet Stockholm Sweden

**Keywords:** Acute intermittent porphyria, case report, heme arginate, pregnancy

## Abstract

In cases of recurrent attacks of acute porphyria during pregnancy, prophylactic administration of heme arginate should be considered. Clinical and biochemical monitoring of the disease and a close collaboration with a porphyria center are crucial.

## Graphical abstract

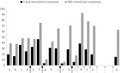

## 1 INTRODUCTION

In cases of recurrent attacks of acute porphyria during pregnancy, prophylactic administration of heme arginate should be considered. Clinical and biochemical monitoring of the disease and a close collaboration with a porphyria expert center are crucial.

Acute intermittent porphyria (AIP) is the most common of the acute hepatic porphyrias, with a European prevalence reported to be approximately 5.4:1 000 000.^1^ Acute intermittent porphyria has a low clinical penetrance, and the incidence of overt disease in European countries is estimated to be 0.13:1 000 000.^1^ Symptoms rarely develop before puberty^2^ and include episodic acute attacks of neurovisceral pain, peripheral neuropathy, hyponatremia, autonomic nervous system dysfunction which, if untreated, can be life‐threatening.

Women are far more likely than men to have clinical manifestations of the disease, and they are usually more seriously affected.^3^, ^4^ Symptoms are associated with endogenous or exogenous fluctuations in progesterone levels—intake of contraceptives, pregnancy, during the luteal phase of their menstrual cycle, when progesterone levels are increased.^3^

Pregnancy involves a unique and unprecedented increase in estrogen and progesterone levels and those hormonal changes can trigger disease manifestations in women with AIP. In the past, pregnancy was associated with a significant morbidity for women with AIP ^5^, ^6^ and there still are numerous case reports of women suffering acute attacks during pregnancy and postpartum.^7^, ^8^ That is also our experience, as a national porphyria reference center.

The current treatment for acute porphyria attacks is the administration of human hemin.^9^ The effects of human hemin on the fetus in utero have not been studied but there are reports of sporadic treatment with human hemin during pregnancy, all with normal outcomes.^5^, ^8^, ^10^ The use of hemin as prophylactic treatment to prevent recurrent severe acute porphyria attacks during pregnancy is not well documented.

## 2 CASE PRESENTATION

**FIGURE 1:**
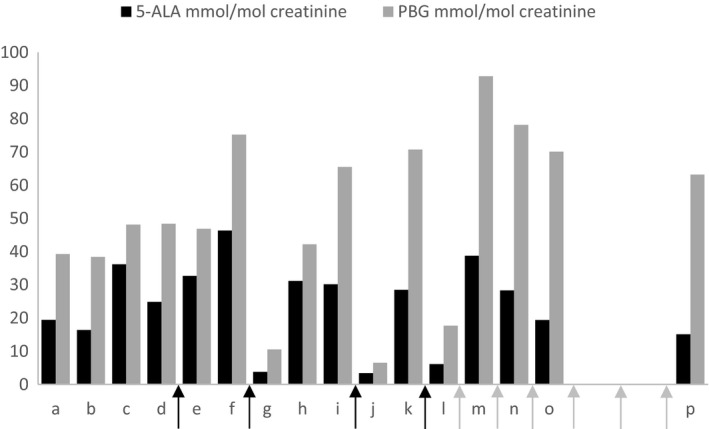
Urinary ALA and PBG levels. a = at diagnosis; b = 1 mo after diagnosis; c = 4 mo after diagnosis; d = hospitalization 6 mo after diagnosis, gestation week 3 + 1; e = gestation week 6 + 6, treatment with iv glucose; f = gestation week 7 + 2, treatment with iv glucose; g = gestation week 7 + 6, directly after heme arginate; h = gestation week 13 + 0; i = gestation week 25 + 0; j = gestation week 26 + 0, 2 d heme arginate; k = gestation week 28 + 5; l = gestation week 30 + 6, 2 d post heme arginate; m = gestation week 33 + 5, 12 d post prophylactic heme arginate; n = gestation week 35 + 0, 10 d post prophylactic heme arginate; o = gestation week 36 + 5, 10 d post prophylactic heme arginate; *P* = 1 d postpartum (partus week 39 + 6), 4 d post prophylactic heme arginate. Arrows indicate treatment with heme arginate: black arrows = treatment for 3‐4 consecutive days, one dose daily, 3 mg/kg; gray arrows = single prophylactic treatment 3 mg/kg. Normal values: 5‐ALA < 3.9 mmoL/moL creatinine, PBG < 1.6 mmoL/moL creatinine

A 25‐year old Caucasian woman was diagnosed with acute intermittent porphyria (AIP). Biochemical testing confirmed an acute porphyria attack, with elevated delta‐aminolevulinic acid (ALA) and porphobilinogen (PBG) in the urine (ALA 19.4 mmoL/moL creatinine, normal values < 3.9 and PBG 39.2 mmoL/moL creatinine, normal values < 1.6, Figure 1). Genetic testing confirmed a pathogenic mutation in the HMBS gene (c.517C>T).^11^ Treatment consisted of intravenous glucose, analgesics (opioids), and laxatives.

Three months after her first attack, the patient was admitted again with clinical and biochemical symptoms indicative of a new attack: abdominal pain, hyponatremia (133 mmol/L−range 137‐145), anxiety, agitation, and constipation. An upper respiratory tract infection was a triggering factor, since infections are an environmental factor that increases hepatic heme biosynthesis and can cause attacks.^12^ Again, treatment consisted of intravenous glucose and opioids.

The disease manifestations intensified after this attack. The patient described daily symptoms (pain, nausea, fatigue, and depression) in her follow‐up visit with her physician. Her urinary ALA and PBG excretion were now chronically elevated. She was hospitalized again shortly after with severe pain, constipation, and slightly elevated serum aminotransferases. Specific treatment with heme arginate (Normosang^©^) 3 mg/kg for three consecutive days was initiated, due to recurrent attacks and to the severity of her symptoms. After the first dose, a pregnancy test was performed at the suggestion of the patient and an early pregnancy (estimated as week 3) was confirmed. The treating physicians consulted with porphyria specialists and it was agreed that the priority in this case would be to treat the porphyria attacks effectively and without delay.

After discharge from the hospital, the patient was still experiencing intensified nausea due to a combination of active porphyria disease and hyperemesis gravidarum. A plan for optimizing nutrition and calorie intake was laid out. Four weeks later, she was admitted with a new porphyria attack. Heme arginate was administered promptly upon admission, 3 mg/kg for four consecutive days. The patient was experiencing severe pain. Antiemetics and opioids were administered on demand (intravenous ketobemidone). She also received parenteral nutrition, considering the combination of early pregnancy, body mass index on the lower normal range (18.9), and debilitating nausea. Her biochemical values (urinary ALA, PBG) responded well to heme arginate (Figure 1) but her symptoms (severe pain requiring opiate treatment, insomnia, nausea, and nutrition difficulties) remained. She was discharged after a month in the hospital, now on regular dosage of oral opiates (oxycontin 10 mg twice daily and ketobemidone 5 mg 2‐3 daily). Her condition was relatively stable for a short period of time, but her intake of opioids increased.

Four months after her second discharge, on gestational week 25, the patient was admitted with a severe acute porphyria attack and was treated with heme arginate 3 mg/kg for four consecutive days. Her ketobemidone dose was at 20‐30 mg daily, and after consulting with a team of pain specialists and specialists in addiction medicine, she was started on selective serotonin reuptake inhibitors, attempting to modulate the pain. The biochemical markers responded to heme arginate treatment (Figure 1). Five weeks later, she was admitted with accentuated pain, nausea, anxiety, and with a significant rebound in her biochemistry (Figure 1). Heme arginate was administered (3 mg/kg for four consecutive days), and this time, the need of prophylactic treatment was assessed. A treatment plan was made to administer heme arginate 3 mg/kg as a single dose in 10‐day intervals starting on gestational week 32,^12^, ^13^ with an adjustment made for the patient's pregnancy weight gain. She received a total of six such prophylactic treatments. Her biochemical status showed chronic high excretion of ALA and PBG (Figure 1) as seen in patients on heme arginate prophylaxis,^13^ but the fluctuations stopped. The treatment inhibited further porphyria attacks. The ketobemidone consumption remained at 30‐35 mg daily, and the assessment by the team of pain/addiction medicine specialists concluded that the opioid intake was due to anxiety and withdrawal symptoms. A plan was set up for tapering and replacement of opiates, postpartum. Since the fetus was exposed to opiates throughout the pregnancy, a high risk of neonatal abstinence syndrome was anticipated.

On gestational week 39 + 6, the patient was induced and had an otherwise uncomplicated vaginal birth. The newborn had an Apgar score of 9‐10‐10, weighed 2900 g, with normal length (48 cm), and head circumference (34 cm) but presented with signs of severe opiate withdrawal syndrome and was therefore treated at the NICU initially. No abnormalities could be seen in his blood tests, liver/kidney function values, there were no signs of iron overload or ferritin increase (values on day 2 after birth, Table 1). He was then reunited with his mother and continued developing normally. The child tested negative for the family's HMBS mutation.

**Table 1:** Biochemical values of newborn, day 2/day 5

Blood/iron indices	Values	Reference	Units
Hemoglobin day 2	184	118‐184	g/L
ALAT day 2	0.55	<1.20	µkat/L
ASAT day 2	1.06	<1.20	µkat/L
Ferritin day 2	387	150‐450	µg/L
Bilirubin day 2	169	<100	µmoL/L
Bilirubin day 5	148	<200	µmoL/L

The total dose of heme arginate that was administered to the patient during her pregnancy is shown on Table 2. The patient was notably anemic throughout her pregnancy and testing pre‐ and postpartum revealed a normal ferritin value (postpartum value 94 µg/L−range 15‐150) and no significant elevation of serum aminotransferases (AST 0.61 µkat/L−range 0.20‐0.60; ALT 0.41 µkat/L−range < 0.75).

**Table 2:** Total amount of heme arginate/iron administered during pregnancy

Gestation week	Heme arginate (mL)	Hemin (mg)
3	21	525
7	28	700
25	28	700
30	28	700
32‐39	60	1500
Total	165	4125
Total iron (mg)		374.5

The child has been developing normally, and there are so far no signs physical or intellectual developmental delay (current age 2.5 years). The patient has continued with prophylactic heme arginate treatment and is now on opiate substitution therapy (methadone).

## 3 DISCUSSION

Restoring hepatic heme biosynthesis through the administration of heme arginate is the treatment of choice in acute intermittent porphyria attacks,^12^ but there are few scientific data on the use of heme arginate during pregnancy. Porphyria expert centers condone the treatment in those cases where the attacks are severe and not responding to supportive treatment with intravenous glucose and the elimination of triggering factors.^5^, ^14^ There are a number or case reports where heme arginate was used during pregnancy with no observations of adverse effects.^7^, ^8^, ^10^ The potential effects on the fetus are not known, and to our knowledge, there is only preclinical data suggesting that heme could cause DNA scission.^15^ General caution has previously been advocated.

In this report, we present a case of a pregnant porphyria patient who experienced recurrent, biochemically verified acute attacks. The treating team initially had a cautious approach to heme arginate, due to the pregnancy and clinical experience indicating that extensive use could result in an increased inflammatory hepatic response.^16^ A collaboration with a porphyria expert center was established early in the pregnancy, and the patient's clinical and biochemical status was closely monitored. By the end of the second trimester, it was obvious that intermittent use of heme arginate to curb attacks failed to keep the porphyria under control. The patient was subsequently treated with prophylactic heme arginate during her third trimester, with satisfactory effect on the clinical manifestations. The patient ceased having attacks for the remainder of her pregnancy. Her over‐excretion of ALA and PBG continued to be manifest during the weekly prophylactic infusions of heme arginate, which is consistent with previous observations.^4^, ^13^ The delivery was uncomplicated, and the newborn had no signs of liver or bone marrow influence or any findings suggesting iron overload. The patient has continued with prophylactic heme arginate treatment. The child is now attending pre‐school and developing normally.

Heme arginate consists of hemin, ferric protoporphyrin IX complex, covalently bound to L‐arginine to prevent rapid degradation. One ampoule of Normosang^®^ (250 mg) contains 22.7 mg iron. Repeated doses of heme arginate have been shown results in iron overload‐related complications.^17^ In this case, neither mother nor child showed signs of iron overload. There were no preexisting conditions that predisposed the patient to iron overload, and the total amount of iron administered in the form of heme arginate is comparable to less than two units of packed red cells.^18^

Prophylactic use of heme arginate, defined as administration of heme arginate on predetermined regular intervals, should be considered in cases of severe recurrent porphyria attacks during pregnancy, when sporadic use is not enough to reverse the disease manifestations. The decision should be made after careful consideration between the patient, her treating obstetrician and a porphyria expert center.

We present a case of a severely afflicted young woman with recurrent porphyria attacks during pregnancy. The decision to treat with heme arginate prophylactically was made late in the pregnancy. The prophylactic treatment inhibited further attacks, did not have any adverse effects in the mother or fetus and should in retrospect have been initiated at an earlier stage.

## CONFLICT OF INTEREST

None declared.

## AUTHORS' CONTRIBUTIONS

DV, ES and PH: provided continuous consultations from the Porphyria Centre regarding the patient's biochemical status and advised on treatment of porphyria‐related symptoms. DV: performed the literature review, analyzed the data, and wrote the manuscript. CL: was responsible for the patient and her child's care and reviewed the manuscript. ES and PH: reviewed the manuscript. All authors: read and approved the final manuscript.

## ETHICAL APPROVAL

The report was approved by the local Ethics Committee of the Karolinska Institutet (Dnr 2018/372‐31), Stockholm.

## CONSENT FOR PUBLICATION

Written informed consent was obtained from the patient and the underage child's both legal guardians for publication of this case report. A copy of the written consent is available for review by the Editor‐in‐Chief of this journal.
